# Acacetin alleviates autoimmune myocarditis by regulating CD4+ T cell mitochondrial respiration

**DOI:** 10.1186/s13020-024-00943-9

**Published:** 2024-05-13

**Authors:** Yang Lu, Yu-Wei Wu, Jiu Pu, Qiong-Feng Wu, Qian Dong, Ning Zhao, Gui-Rong Li, Yi-Mei Du

**Affiliations:** 1 Department of Cardiology, Union Hospital, Tongji Medical College, Huazhong University of Science and Technology, Wuhan, China; 2 Research Center of Ion Channelopathy, Union Hospital, Tongji Medical College, Huazhong University of Science and Technology, Wuhan, China; 3 Institute of Cardiology, Union Hospital, Tongji Medical College, Huazhong University of Science and Technology, Wuhan, China; 4 Key Lab for Biological Targeted Therapy of Education Ministry and Hubei Province, Union Hospital, Tongji Medical College, Huazhong University of Science and Technology, Wuhan, China; 5 Hubei Provincial Engineering Research Center of Immunological Diagnosis and Therapy for Cardiovascular Diseases, Union Hospital, Tongji Medical College, Huazhong University of Science and Technology, Wuhan, China; 6 Department of Cardiology, The First Affiliated Hospital of Zhengzhou University, Zhengzhou, China; 7 Nanjing Amazigh Pharma Limited, Nanjing, Jiangsu China

**Keywords:** Acacetin, Myocarditis, CD4+ T cells, Th17 cells, Mitochondrial complex II, Mitochondrial respiration

## Abstract

**Background:**

Myocarditis refers to an autoimmune inflammatory response of the myocardium with characterization of self-reactive CD4+ T cell activation, which lacks effective treatment and has a poor prognosis. Acacetin is a natural flavonoid product that has been reported to have anti-inflammatory effects. However, acacetin has not been investigated in myocarditis.

**Methods:**

Oral acacetin treatment was administered in an experimental autoimmune myocarditis model established with myosin heavy chain-alpha peptide. Echocardiography, pathological staining, and RT-qPCR were used to detect cardiac function, myocardial injury, and inflammation levels. Flow cytometry was utilized to detect the effect of acacetin on CD4+ T cell function. RNA-seq, molecular docking, and microscale thermophoresis (MST) were employed to investigate potential mechanisms. Seahorse analysis, mitoSOX, JC-1, and mitotracker were utilized to detect the effect of acacetin on mitochondrial function.

**Results:**

Acacetin attenuated cardiac injury and fibrosis as well as heart dysfunction, and reduced cardiac inflammatory cytokines and ratio of effector CD4+ T and Th17 cells. Acacetin inhibited CD4+ T cell activation, proliferation, and Th17 cell differentiation. Mechanistically, the effects of acacetin were related to reducing mitochondrial complex II activity thereby inhibiting mitochondrial respiration and mitochondrial reactive oxygen species in CD4+ T cells.

**Conclusion:**

Acacetin may be a valuable therapeutic drug in treating CD4+ T cell-mediated myocarditis.

**Graphical Abstract:**

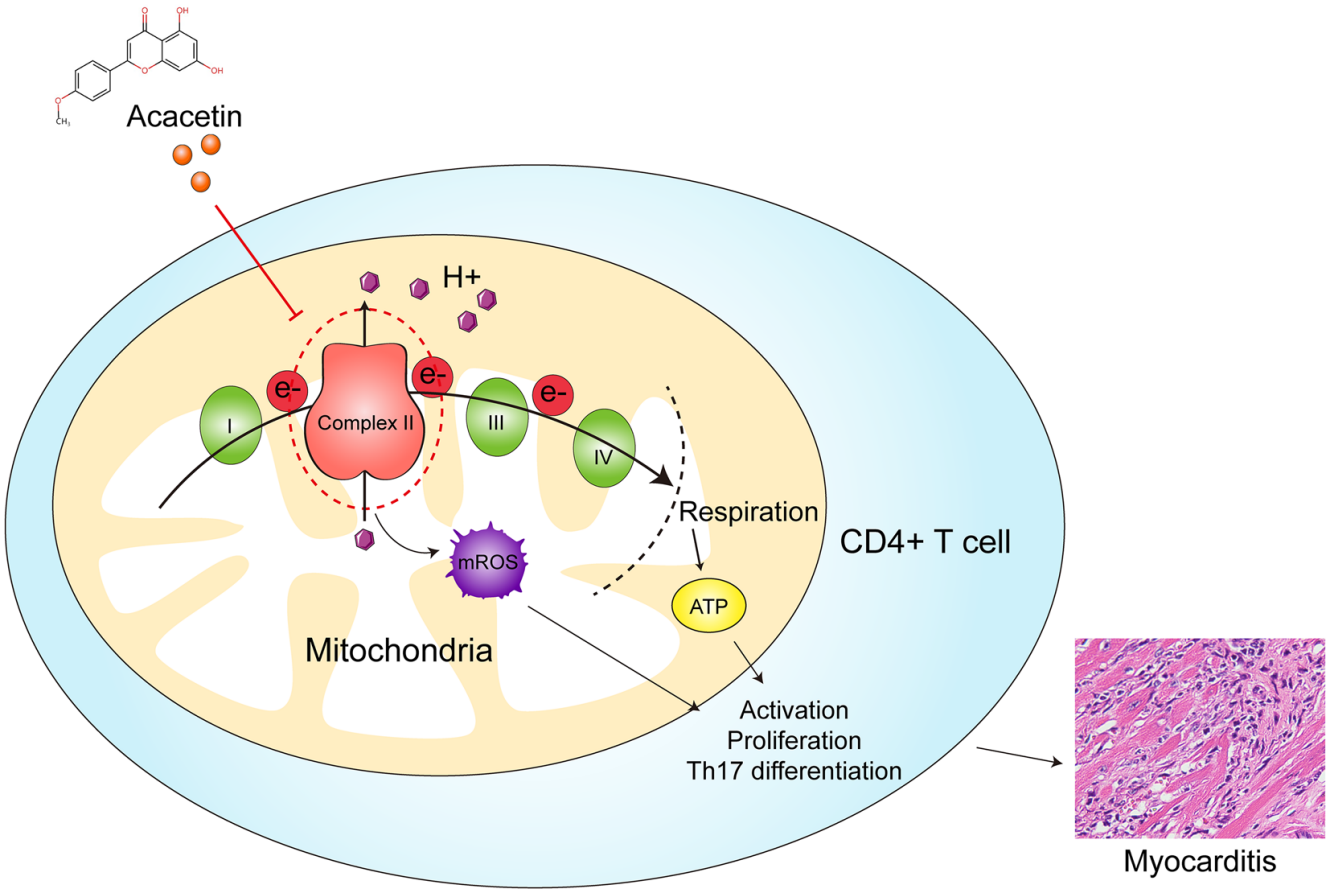

**Supplementary Information:**

The online version contains supplementary material available at 10.1186/s13020-024-00943-9.

## Introduction

Myocarditis is an autoimmune inflammatory disease characterized by myocyte necrosis and inflammatory cell infiltration. It is a common cause of sudden death in adolescents and usually progresses to dilated cardiomyopathy (DCM) and heart failure, resulting in a poor prognosis. Clinical treatments of myocarditis are usually supportive, which include corticosteroid therapy (to help reduce inflammation), cardiac medications, such as a beta-blocker, ACE inhibitor, a low-salt diet, diuretic therapy to treat fluid overload, antibiotic therapy, etc. [1]. Recent studies show that activation of self-reactive effector CD4+ T cells is a main driver that causes myocardial injury and progression of DCM [2], such as IL-17-producing Th17 cells [3, 4]. The effector CD4+ T cells exacerbate myocarditis by promoting viral replication, secretion of cytokines, myeloid cell infiltration, and myocardial fibrosis [5]. Therefore, targeting CD4+ T cell activation may be crucial for developing new treatment of myocarditis.

The natural flavone acacetin (5,7-dihydroxy-4-methoxyflavone) has been reported to have antioxidant and anti-inflammatory properties. Recent studies have experimentally shown that acacetin is effective in several cardiac diseases such as atrial fibrillation, myocardial infarction, and diabetes cardiomyopathy [6–8]. Acacetin inhibits oxidative stress during myocardial ischemia/reperfusion injury by activating Nrf-2 [9] and alleviating cardiac senescence by targeting mitochondrial autophagy [10]. We previously demonstrated that acacetin inhibits human T cell activation via blocking Kv1.3 channels [11]. However, the detailed mechanism of acacetin action on T cells and its role in the pathogenesis of myocarditis remain unclear.

In the present study, we established an experimental autoimmune myocarditis (EAM) model in BALB/c mice and evaluated the effects of acacetin on cardiac injury and heart function and CD4+ T cell-associated immune responses in EAM mice. We analyzed the effects of acacetin on CD4+ T cell activation and proliferation. RNA sequencing (RNA-seq) molecular docking, and microscale thermophoresis (MST) were used to evaluate the mechanism of acacetin’s action on CD4+ T cells. Finally, we examined if acacetin could prevent the progression of DCM. Acacetin appears to be a promising therapeutic drug for myocarditis treatment.

## Materials and methods

### Experimental animals

Six- to seven-week-old male BALB/c mice were purchased from SPF Biotechnology Ltd (Beijing, China) and maintained in an SPF facility at Tongji Medical College. Animal experimental protocols were approved by the Animal Care Committee of the Union Hospital of Tongji Medical College, Huazhong University of Science and Technology ([2022] IACUC Number 3029). The experimental procedure followed the guidelines from Directive 2010/63/EU of the European Parliament on the protection of animals used for scientific purposes.

### EAM model induction and drug treatment

EAM model was established in BALB/c mice with cardiac-specific peptide-α (α-MHC: Ac-SLKLMATLFSTYASAD-OH) purchased from Shanghai GL Biochemical Company. The peptide was dissolved in saline and emulsified with complete Freund’s adjuvant at a 1:1 ratio. On day 0 and day 7, 0.2 ml of the emulsion was injected subcutaneously into the animals. Experimental animals were divided into three groups: the control group, the EAM + vehicle group (Vehicle group), and the EAM with acacetin treatment group (Acacetin group).

Acacetin used in the present study was synthesized in the laboratory as described previously [7]. EAM mice receiving drug treatment were gavaged with suspension solution of acacetin (100 mg/kg daily) with hydroxypropyl-β-cyclodextrin, while control mice and vehicle mice were gavaged with equivolume of hydroxypropyl-β-cyclodextrin solution for 3 weeks or 2 weeks as specifically described in the results.

### Echocardiography

Echocardiography was performed using a Vevo 1100 ultrasound system (Visual Sonics, Toronto, Canada) to evaluate heart function in mice anesthetized with 1.5% isoflurane to obtain two-dimensional views of the short mid-ventricular axis and the long parasternal axis. Heart function was analyzed in mice using Vevo LAB software.

### Histopathology

Cardiac ventricular tissues were fixed in 4% formalin, embedded in paraffin, and sectioned. Traditional hematoxylin/eosin (H&E) and Masson’s trichrome staining were used to determine myocardial inflammation and fibrosis, respectively. Inflammation was scored using the methods as previously described [12]. Myocardial fibrosis was measured by Image-Pro Plus software as a proportion of the area of blue staining in the section slices. Two independent observers assessed the section slices, anonymizing them to minimize potential bias.

### Cell isolation and culture

Naive CD4+ T cells were isolated from spleens and lymph nodes of normal mice using the Naive CD4+ T Cell Isolation Kit (STEMCELL, Vancouver, Canada) and cultured with plates pre-coated with anti-mouse CD3/CD28 (both 5 μg/mL, Biolegend, San Diego, CA, USA) for 2–4 h. Cells were incubated in IMDM medium supplemented with 10% FBS (Sciencell, Vancouver, Canada), 2 mM l-glutamine (Gibco, New York, USA), 0.1 mM non-essential amino acids (Gibco, USA), 1% penicillin/streptomycin (Servicebio, Wuhan, China), and 55 μM β-mercaptoethanol (Gibco, New York, USA). For Th17 cell differentiation, 50 ng/mL IL-6, 2.5 ng/mL TGF-β, 5 μg/mL anti-IFN-γ, and 5 μg/mL anti-IL-4 (Biolegend, San Diego, CA, USA) were added to the medium. Acacetin was solubilized with DMSO and added to the medium at beginning of culture in the absence or presence of dimethyl succinate (DES, 5 mM, MedChemExpress, Shanghai, China) and Atpenin A5 (1 μM or 2 μM, MedChemExpress). Fluorescence was detected by flow cytometry on second or 3rd day of the culture.

### Flow cytometry

The flow cytometry was used to analyze cell populations by incubating cells with different antibodies or fluorescence. The single cell suspensions were incubated with anti-CD16/32 (Biolegend, San Diego, CA, USA) prior to staining to block non-specific binding to the Fc receptor. Cells were incubated for 30 min at 4 ℃ for surface staining. For intracellular cytokine staining, cells were incubated with a stimulating/blocking cocktail for 4 h prior to surface staining and cytokine staining was performed after treatment with fixation/permeabilization kits.

The following antibodies were used: FITC anti-CD45, APC-Cy7 anti-CD11b, Brilliant Violet 421 anti-Ly6C, PE anti-CD44, PE anti-F4/80, APC anti-Ly6G, APC anti-CD62L, APC anti-CD25, PE anti-IL-17A, and Zombie Aqua Fixable Viability Kit (Biolegend, San Diego, CA, USA).

For CFSE staining, cells were incubated with 1.25 μM CFSE (Biolegend, San Diego, CA, USA) for 10 min prior to incubation. For mitochondrial reactive oxygen species (mROS) assay, cultured cells were incubated with 5 μM MitoSOX (Invitrogen, Carlsbad, USA) for 30 min. For mitochondrial membrane potential (MMP) assay, cultured cells were incubated with JC-1 (1:200, Biyuntian, Wuhan, China) in buffer for 10 min. For mitochondrial mass assay, cultured cells were incubated with 5 μM mitoTracker (Invitrogen, Carlsbad, USA) for 30 min. Fluorescence was measured by flow cytometry.

### Real-time quantitative PCR (RT-qPCR)

Total RNA from myocardial tissues or cultured CD4+ T cells was extracted using TRIzol reagent (Invitrogen, Carlsbad, USA) and 1000 ng of RNA was reverse transcribed using the PrimeScript RT kit (Vazyme, Nanjing, China) to generate cDNA. The related primers (Table S1) were purchased from Tsingke (Beijing, China). Data analysis was performed using the 2^−ΔΔCT^ method. The amplified specific cDNA was quantified using AceQ Universal SYBR qPCR Master Mix (Vazyme, Nanjing, China) in a Bio-Rad CFX CONNECT detector system.

### RNA-seq analysis

Total RNA was isolated from cultured CD4+ T cells on day 3 using TRIzol reagent (Invitrogen, Carlsbad, USA) according to the manufacturer’s protocol. Libraries were prepared using TruSeqTM RNA Sample Preparation Kit (Illumina, San Diego, CA). The mRNA was reverse transcribed into cDNA using the SuperScript double-stranded cDNA synthesis kit, and then sequenced using Illumina HiSeqxten/NovaSeq 6000. The quality of raw and trimmed Fastq reads was checked using Hisat2. The raw data were trimmed using the Stringtie tool. The R package “DESeq2” was used to analyze differentially expressed genes between two groups. Genes with an absolute value of fold change > 1.5 and adjusted p-value < 0.05 were considered differentially expressed. The R package “clusterProfile” was used to implement the GSEA algorithm using c2 (c2.cp.v7.5.1.symbols.gmt) in the Molecular Signature Database to analyze changes in pathways between two groups. All sequencing data are available through the NCBI Gene Expression Omnibus (GEO) database under accession number  GSE221244.

### Molecular docking

The compound names, molecular weights, and three-dimensional (3D) structures of acacetin were obtained from the PUBCHEM database. 3D structures of Complex I-V were obtained from the Protein Data Bank database (Complex I: 5XTD, Complex II: 3AEF, Complex III: 5XTE, Complex IV: 5Z62, Complex V: 6J5J). Subsequently, the ligands and proteins required for molecular docking were prepared using AutoDock Vina software (http://vina.scripps.edu/). Finally, the docking results were analyzed by AutoDock tools and PyMOL. The binding ability of acacetin and Complex I-V was assessed by the affinity (kcal/mol) values.

#### MST assay

In the streamlined protocol for detecting the binding of SDHA protein to acacetin using MST, the process begins by preparing a dye solution and adjusting the SDHA protein (Solarbio, #P08777) concentration to 200 nM. The protein is then labeled by mixing with the dye solution and incubating for 30 min at room temperature, followed by centrifugation to remove aggregates. For the binding assay, acacetin is serially diluted and mixed with the labeled protein in wells, aiming for a final protein concentration of 50 nM. Ligand concentrations range from 100 μM to 3.05 nM. The MST analysis is performed at medium power and 50% LED intensity at 25 ℃. Data analysis, done with MO.Affinity Analysis Software v2.3, employs a one-site binding model to calculate the dissociation constant (KD) for the SDHA-acacetin interaction, providing insight into their binding affinity.

#### Seahorse mitochondrial respiration rate assay

Mitochondrial respiration rate was assessed by the Mito Stress Test Kit (Agilent, Santa Clara, CA, USA) and the Seahorse Bioscience XF24 Extracellular Flux Analyzer (Agilent, Santa Clara, CA, USA). Cells were inoculated into Seahorse XF 24-well culture plates (500,000 cells per well). The assay medium (supplemented with 10 mM glucose, 1 mM pyruvate, 2 mM glutamine, and 5 mM HEPES) was then stabilized in a CO_2_-free incubator at 37 ℃ for 1 h. After baseline measurements, oligomycin, FCCP, and rotenone/antimycin A were added sequentially to detect oxygen consumption rate.

#### Statistical analysis

Statistical analyses were performed using GraphPad Prism 9.0 software. One-way ANOVA followed by Tukey’s post hoc test or Kruskal–Wallis with Dunn’s multiple comparisons test was used for multiple group comparisons. Unpaired two-tailed Student's t-test was used to compare two groups. Data are presented as mean ± SD. P-value < 0.05 was considered statistically significant.

## Results

### Acacetin ameliorates heart dysfunction and cardiac injury in EAM mice

**Fig. 1:**
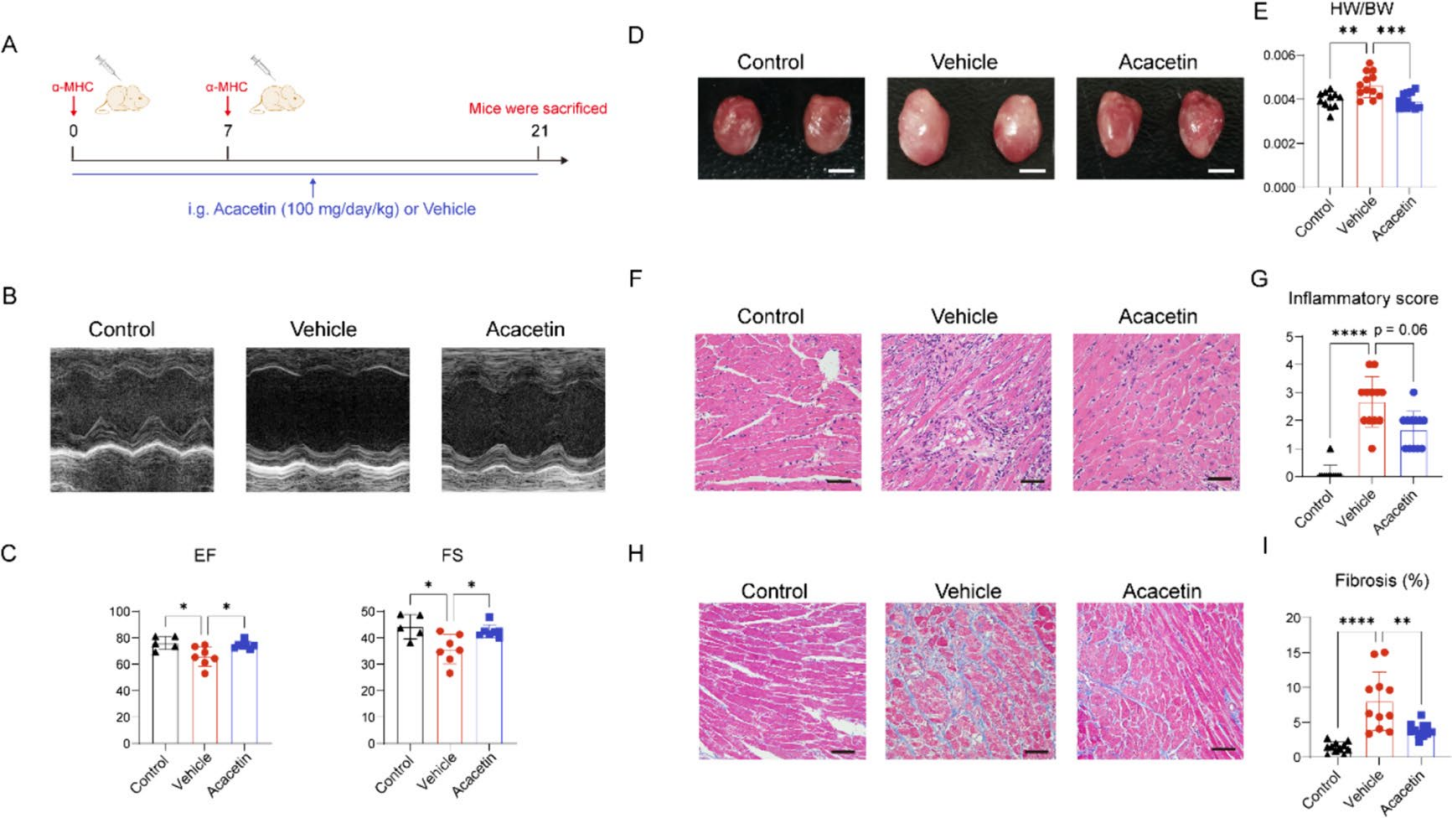
Acacetin attenuates myocardial injury in EAM mice. **A** Animal experiment protocol, α-MHC (0.2 mL) was subcutaneously injected at day 0 and day 7, and acacetin (100 mg/kg daily) or vehicle was orally administrated from day 0 to day 21 of the experiment. **B** Representative images of echocardiography in control mouse, EAM mouse (vehicle), and EAM mouse treated with acacetin. **C** Percent values of left ventricular EF and FS measured with echocardiography in animals as in B (n = 5–7). **D** Representative heart images from control nice, EAM model mice, and EAM mice treated with acacetin. Scale bar = 5 mm. **E** HW/BW for each group (n = 11–12). **F** Representative HE-stained images for analyzing inflammation score in ventricular section slices from mice as in **B**. **G** Mean values of inflammatory score in myocardial tissues in mice as in **B** (n = 11–12). Scale bar = 50 μm. **H** Representative images of Masson staining in ventricular section slices from mice as in **B**. **I** Mean percent values of quantified fibrotic area in mice as in **B** (n = 11) for each group. Scale bar = 50 μm. Data are expressed as mean ± SD and analyzed with one-way ANOVA followed by Tukey’s post hoc test or Kruskal–Wallis with Dunn multiple comparisons test. *P < 0.05, **P < 0.01, ***P < 0.001, or ****P < 0.0001

The potential protective effect of acacetin against myocarditis was assessed in a mouse model of EAM established by injection of myosin heavy chain-α peptides [13]. The EAM mice with drug treatment were gavaged with acacetin suspension (100 mg/kg daily) from day 0 to day 21 (Acacetin group), while EAM mice gavaged with equivolume vehicle were used as positive controls for disease (Vehicle group, Fig. 1A). Heart function was evaluated at end of experiment (day 21) using echocardiography in anesthetized mice (Fig. 1B). Significant reductions in left ventricular ejection fraction (EF) and fractional shortening (FS) were observed in the Vehicle group (n = 5–7, P < 0.01 vs Control group). However, these decreased EF and FS were reversed in the Acacetin group (Fig. 1C, Table 1). These results suggest that acacetin improves heart dysfunction in EAM mice.

**Table 1:** Echocardiographic parameters in mice at day 21 of experiments

	Control	Vehicle	Acacetin
Heart rate (bpm)	433.3 ± 94.67	432.3 ± 61.50	448.9 ± 80.23
EF (%)	76.05 ± 5.06	65.84 ± 7.42^*^	74.43 ± 2.79^#^
FS (%)	44.21 ± 4.58	35.72 ± 5.46^*^	42.45 ± 2.51^#^
LV end-diastolic diameter	3.61 ± 0.24	3.37 ± 0.19	3.33 ± 0.21
LV end-systolic diameter	2.02 ± 0.26	2.17 ± 0.23	1.91 ± 0.16

Data show mean ± SD. P values were determined by one-way ANOVA followed by Tukey’s post hoc test

*EF* ejection fraction, *FS* fractional shortening, *LV* left ventricle

*P < 0.05 compared to Control, #P < 0.05 compared to Vehicle

Following echocardiographic analysis, the hearts were isolated from the anesthetized animals. The heart size was clearly greater in EAM model animals compared with control or acacetin-treated EAM animals (Fig. 1D). The ratio of heart weight to body weight (HW/BW) was increased in EAM mice administered with vehicle and normalized in those treated with acacetin. Subsequently, ventricular sections of mouse hearts were prepared and stained with HE (Fig. 1F) or Masson (Fig. 1H). Evident signs of inflammatory injury and fibrosis were observed in the hearts of EAM animals, which were ameliorated in those treated with acacetin. The increased inflammatory score (Fig. 1G) and fibrosis percentage area (Fig. 1I) in EAM hearts were significantly reduced in EAM with acacetin treatment. Collectively, these findings suggest that acacetin attenuates myocardial injury and fibrosis in EAM mice.

### Acacetin inhibits immune response of autoimmune myocarditis

**Fig. 2:**
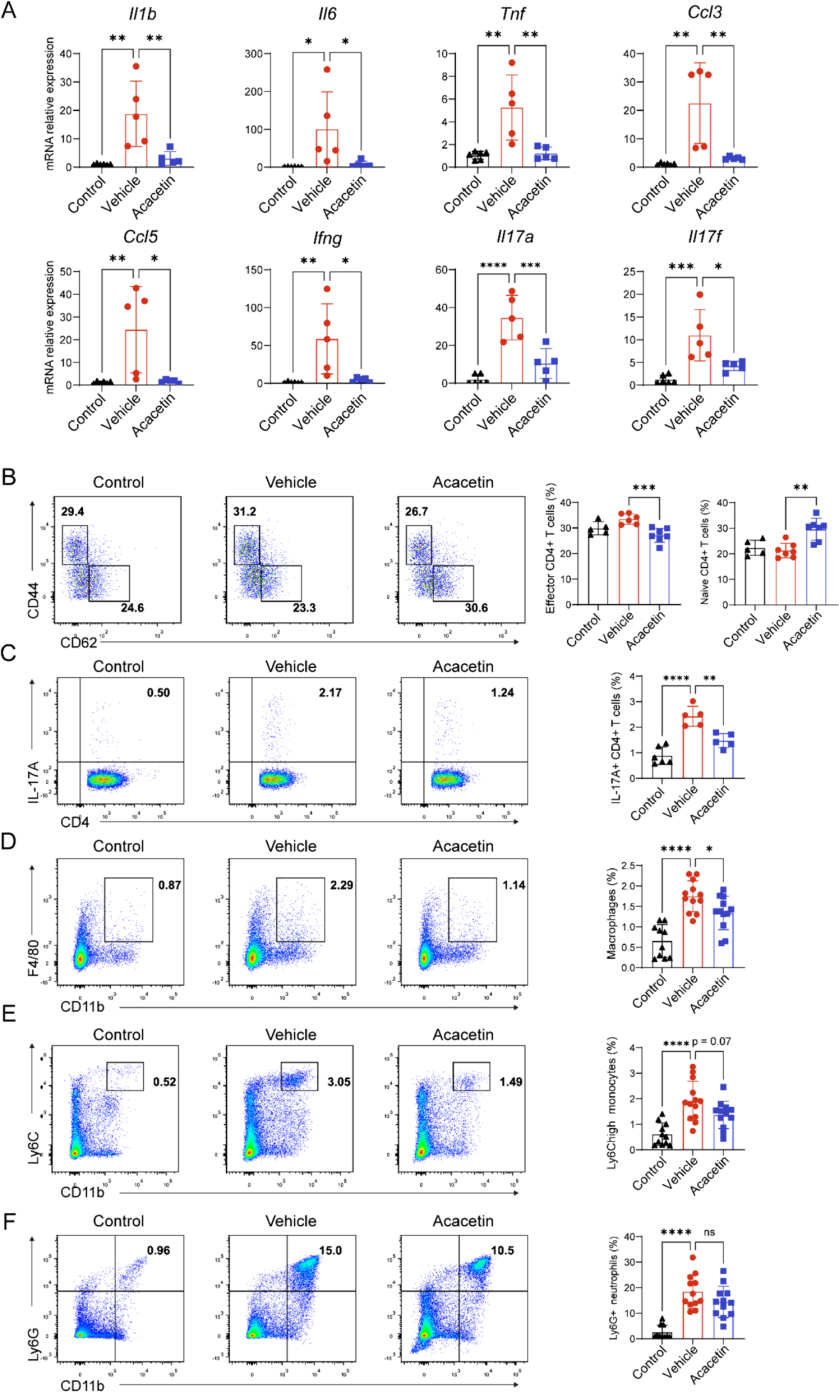
Acacetin decreases inflammatory cytokines or cells in EAM mice. **A** Mean values of the gene expression of *Il1b*, *Il6*, *Tnf*, *Ccl3*, *Ccl5*, *Ifng*, *Il17a*, *Il17f* in ventricular tissues from control mice, EAM mice with vehicle and EAM mice with acacetin (n = 5–6, relative to GAPDH). **B**–**F** Representative flow cytometry graphs of CD4+ T cells and percent values of naïve CD4+ T cells and effector CD4+ T cells (**B**, n = 5–7), Th17 cells with expressing IL-17A (**C**, n = 5–7), macrophages (**D**), Ly6C.^high^ monocytes (**E**), neutrophils (**F**) in cells of spleens from mice as in **A** (n = 5–12). Data are mean ± SD and analyzed with one-way ANOVA followed by Tukey’s post hoc test. *P < 0.05, **P < 0.01, ***P < 0.001, or ****P < 0.0001

The expression levels of inflammatory cytokines were assessed using RT-qPCR in myocardial tissues from each experimental group. The pro-inflammatory cytokines *Il1b*, *Il6*, and *Tnf* as well as the chemokines *Ccl3* and *Ccl5* were remarkably enhanced in ventricular tissues of EAM mice with vehicle (Fig. 2A), these increases were reversed in EAM mice treated with acacetin. Similarly, the expression levels of CD4+ T cell and Th17 cell effector cytokines, including Ifng, Il17a, and Il17f, were upregulated in myocardial tissues of EAM mice administered with vehicle (see Fig. 2A), but were downregulated in acacetin-treated EAM (Fig. 2A). These findings suggest that acacetin suppresses the production of myocardial inflammatory factors in EAM mice.

To determine the correlation of acacetin protection against EAM to the effector CD4+ T and Th17 cell-mediated immunity involvement, spleen cells were isolated from different groups and analyzed with flow cytometry for the effector CD4+ T cells (CD62L- CD44+ CD4+ cells), naive CD4+ T cells (CD62L+ CD44- CD4+ cells), Th17 cells, myeloid cells (e.g. monocytes, macrophages, and neutrophils), etc. The proportion of effector CD4+ T cells was elevated in EAM mice administered with vehicle, but this increase was attenuated in EAM mice treated with acacetin. The proportion of naive CD4+ T cells remained unchanged in EAM mice administered with vehicle, while it was increased in EAM mice treated with acacetin (Fig. 2B). The percentage of Th17 cells was remarkably increased in EAM mice with vehicle and significantly reversed in EAM mice with acacetin (Fig. 2C). In addition, acacetin treatment significantly decreased the increased proportion of macrophages (Fig. 2D) in EAM with vehicle, but not the elevated percent values of Ly6C^high^ inflammatory monocytes (Fig. 2E, P = 0.07) and neutrophils (Fig. 2F, P = ns). These findings suggest that acacetin’s protective effects against EAM may be attributed to its ability to inhibit CD4+ T cell activation and Th17 cell differentiation in EAM mice.

### Acacetin inhibits CD4+ T cell activation, proliferation, and Th17 cell differentiation *in ex-vivo*

**Fig. 3:**
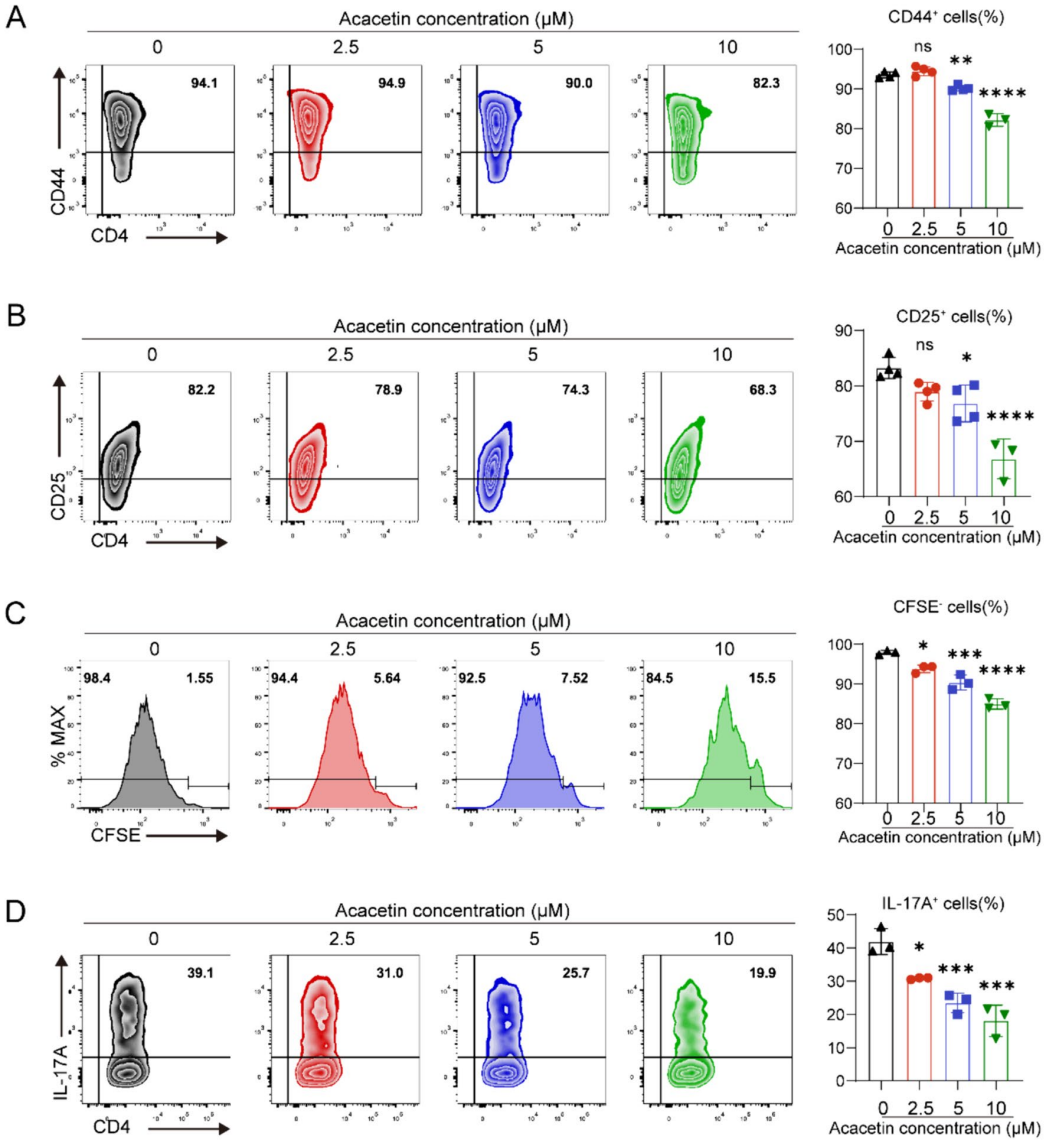
Acacetin inhibits T cell activation, proliferation and Th17 cell differentiation in ex-vivo. **A**–**C** Flow cytometry graphs of T cells isolated from spleen and cultured with anti-CD3/CD28 in the absence and presence of 2.5, 5 or 10 μM for 72 h and the percent values of CD44 (**A**), CD25 (**B**), and proliferative capacity (**C**) (n = 3–4). **D** Flow cytometry graphs and percent values of Th17 cells (expressing IL-17A), determined in naive CD4+ T cells activated in ex-vivo with anti-CD3/CD28 under Th17-polarizing conditions the absence and presence of different concentrations of acacetin for 72 h (n = 3). Data are expressed as mean ± SD and analyzed with one-way ANOVA followed by Tukey’s post hoc test. *P < 0.05, **P < 0.01, ***P < 0.001, or ****P < 0.0001 compared to vehicle (0 μM acacetin)

The effects of acacetin on the activation, proliferation, and Th17 differentiation of CD4+ T cells were investigated using naive CD4+ T cells isolated from the spleens of normal mice. These cells were stimulated with anti-CD3/CD28 antibodies in the presence or absence of acacetin at concentrations of 2.5, 5, or 10 μM. A preliminary experiment revealed no alterations in the viability of naive CD4+ T cells following treatment with acacetin (Figure S1). However, treatment with acacetin at concentrations of 5 and 10 μM markedly decreased the percentage of cells expressing CD44 and CD25 (markers of T cell activation; Fig. 3A and B). Moreover, acacetin inhibited T cell proliferation in a dose-dependent manner (Fig. 3C). Notably, acacetin suppressed Th17 cell differentiation (Fig. 3D). These results suggest that acacetin directly inhibits CD4+ T cell activation, proliferation, and Th17 cell differentiation.

### RNA-seq reveals the effects of acacetin on transcriptomic profile of CD4+ T cells

**Fig. 4:**
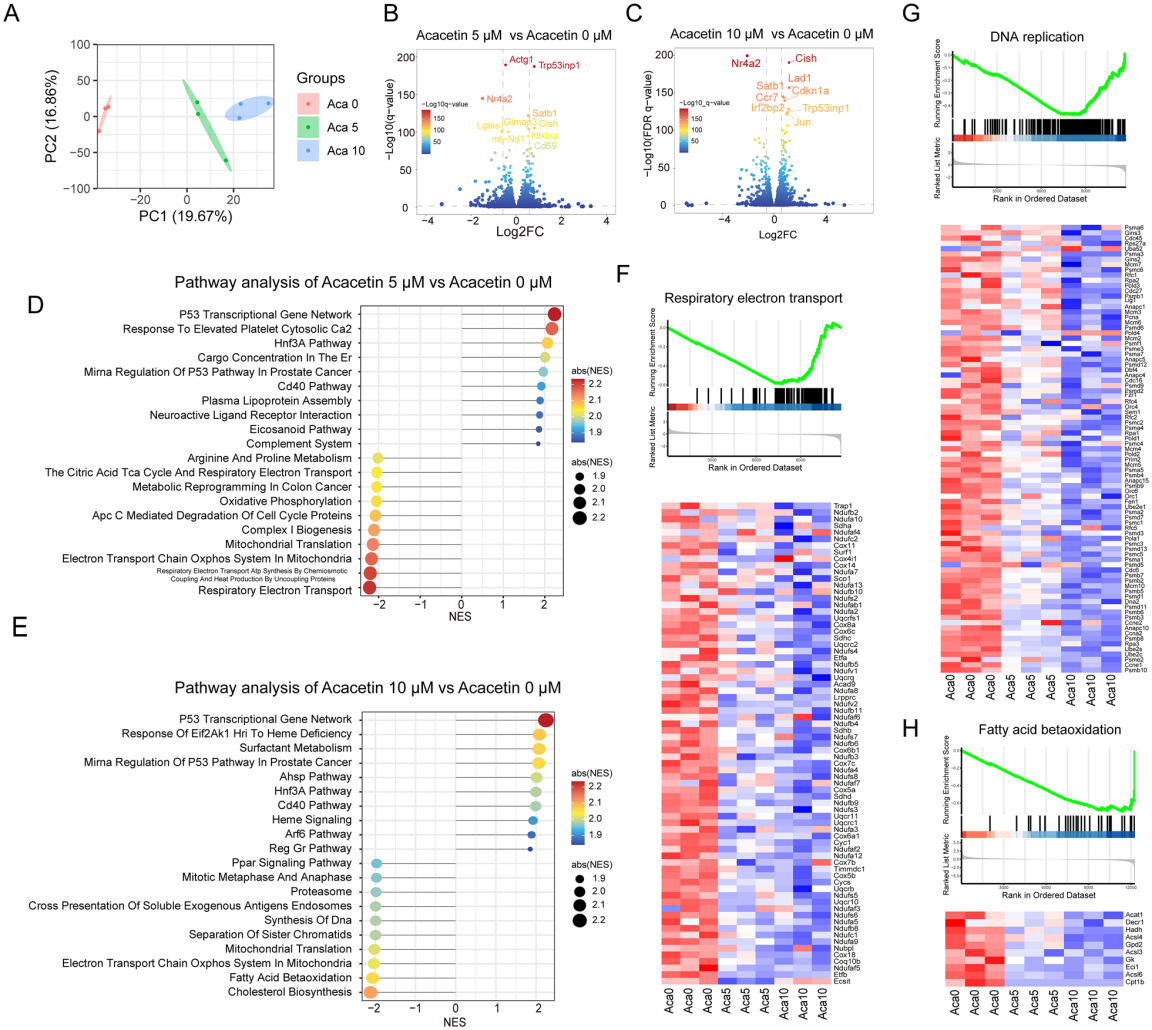
RNA-seq reveals the transcriptional alterations in cultured CD4+ T cells treated with acacetin. **A** Principal Component analysis (PCA) graph of global gene expression in naive CD4+ T cells activated with anti-CD3/CD28 and cultured in the absence and presence of 0, 5, or 10 μM acacetin for 72 h. **B** and **C** Volcano plots showing the difference in gene expression in **B** (5 vs 0 μM acacetin) and **C** (10 vs 0 μM acacetin). The colors of the dots correspond to the magnitude of the (-log_10_ q.value), and genes in the top 20 of the (-log_10_ q.value) are labeled. **D** and **E**. GSEA algorithm using c2 (c2.cp.v7.5.1.symbols.gmt) in the Molecular Signature Database to analyze pathway changes in **D** (5 vs 0 μM acacetin) and **E** (10 vs 0 μM acacetin). **F** GSEA map and gene expression heat map of the respiratory electron transport pathway (5 vs 0 μM acacetin). **G**: Gene expression heat map of the DNA replication. **H** GSEA map and gene expression heat map in the fatty acid bio-oxidation pathway (10 vs 0 μM acacetin). *Aca* acacetin

The RNA-seq was employed to examine the effects and potential mechanisms of acacetin on CD4+ T cells treated with 5 or 10 μM acacetin by analyzing the transcriptomic profile. Principal Component Analysis (PCA) shows that the spatial distance of the cells with 5 μM and 10 μM acacetin treatment was close, but far from the cells without acacetin, demonstrating that acacetin caused a significant alteration in the CD4+ T cell transcriptome (Fig. 4A). The differentially expressed genes were then analyzed in cells in the presence of 0, 5 or 10 μM acacetin. A total of 157 genes were up-regulated and 158 genes were down-regulated in cells with 5 μM acacetin compared with 0 μM acacetin (Fig. 4B). Meanwhile, there were 428 genes up-regulated and 336 genes down-regulated in cells with 10 μM (Fig. 4C). The volcano plot shows 20 genes with the smallest adjusted-P values. We found that a significant proportion of differentially expressed genes (277) at 5 μM were also present at 10 μM (Figure S2A, Table S2). Conversely, 487 genes emerged as differentially expressed exclusively at the 10 μM concentration (Figure S2A, Table S2). This suggests that these genes represent variations specific to the higher concentration.

Afterward, changes in the potential pathways were analyzed using the GSEA algorithm. The GSEA analysis revealed that 5 or 10 μM acacetin treatment induced a decrease in cell cycle-related pathways, e.g. APC/C-mediated degradation of cell cycle proteins, segregation of sister chromatids, and synthetic of DNA (Fig. 4D and E), which is consistent with the inhibition of cell proliferation (Fig. 3C). In addition, 5 μM treatment acacetin induced a significant decrease in mitochondrial respiration-related pathways, e.g. electronic transport of the respiratory system and electron transport chain OXPHOS system in mitochondria (Fig. 4D). 10 μM acacetin treatment resulted in a significant decrease in lipid metabolism-related pathways, e.g. cholesterol biosynthesis and fatty acids beta oxidation, as well as reduction in electron transport chain OXPHOS system in mitochondria, proteasome, and PPAR signaling pathways (Fig. 4E). GSEA and gene expression heatmap also revealed that acacetin decreased the expression of genes related to the cell cycle, the electron transport, and fatty acids beta oxidation (Fig. 4F–H). In addition, GO analysis of common DEGs by both concentrations showed pathways including positive regulation of cytokine production, protein localization to mitochondrion, and cell development, similar to the results of GSEA analysis (Figure S2B). In comparison, the specific pathway at the 10 μM concentration was based on the process of actin filaments, protein serine/threonine kinase activity, and T-cell differentiation (Figure S2B), which may reflect a further inhibition of mitochondrial function. In summary, these results indicate that acacetin mainly affects the cell cycle, mitochondrial respiration, and lipid metabolism in CD4+ T cells.

### Acacetin reduces mitochondrial respiration in T cells

Previous research has demonstrated the critical role of mitochondrial respiration in T-cell activation, proliferation, and differentiation. Diminished mitochondrial respiration hinders lipid metabolism and affects DNA and nucleic acid synthesis [14, 15]. GSEA analysis and heatmap showed that acacetin 5 μM caused an extensive inhibition of mitochondrial respiratory pathway (Fig. 4F). This suggests that acacetin first interferes mitochondrial function and thereby affects lipid metabolism and cell cycle of T cells. The heatmap revealed that most of the downregulated genes were subunits of electron transport chain complexes I, II, III, IV, and V, which are involved in mediating mitochondrial respiration and OXPHOS (Fig. 4D-H). RT-qPCR further confirmed the decrease in mitochondrial respiration and genes related to oxidative phosphorylation following acacetin treatment (Figure S3). These findings indicate that acacetin may primarily target mitochondrial energy metabolism in CD4+ T cells.

**Fig. 5:**
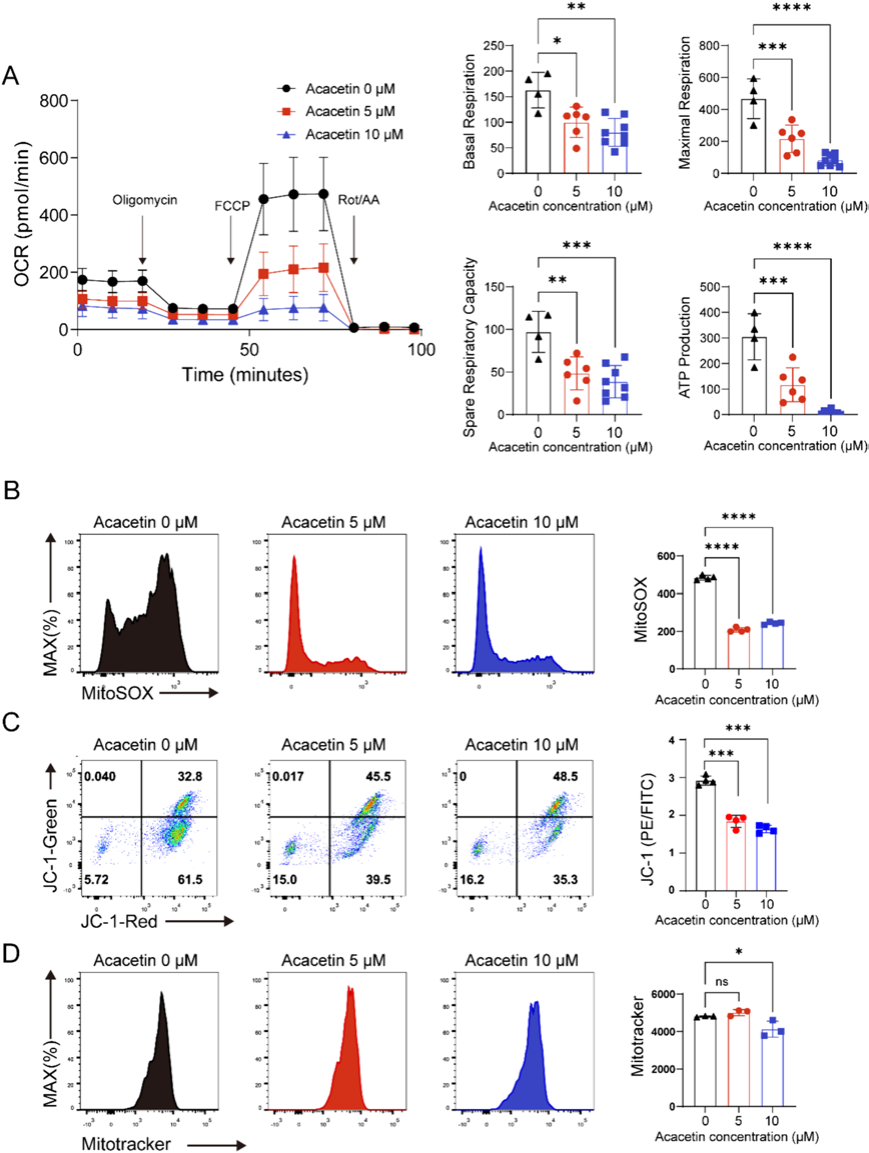
Acacetin inhibits mitochondrial respiration rate, ROS production and membrane potential in CD4+ T cells. **A** Mean values of oxygen consumption rates (OCRs, measured by extracellular flux analysis), basal respiration, spare respiratory capacity, and ATP production in naive CD4 + T cells activated with anti-CD3/CD28 and cultured in the absence and presence of 0, 5 or 10 μM acacetin for 48 h (n = 4–8). **B** Flow cytometry graphs and mean levels of mROS in cells treated as in A and loaded with MitoSOX. **C** Flow cytometry graphs and mean levels of MMP in cells treated as in A and loaded with JC-1. **D** Flow cytometry graphs and mean levels of mitochondrial mass in cells treated as in A and loaded with mitoTracker (n = 3–4). Data are expressed as mean ± SD and analyzed with one-way ANOVA followed by Tukey’s post hoc test. *P < 0.05, **P < 0.01, ***P < 0.001, or ****P < 0.0001

The impact of acacetin on regulating mitochondrial respiration was further explored by assessing the OCR of T cells using the Seahorse Cell Mito Stress Test. Acacetin (5 or 10 μM) significantly inhibited OCR, basal and maximal respiration, reserve respiratory capacity, and ATP production in T cells following the uncoupling of mitochondria from FCCP (Fig. 5A). These results suggest that acacetin primarily inhibits respiratory reserve, thereby limiting the energy available for T cell activation.

### Acacetin decreases mROS production and membrane potential in CD4+ T cells

It is widely accepted that mitochondrial reactive oxygen species (mROS) generated by the oxidative respiratory chain promote intracellular signaling, thereby enhancing T cell activation and proliferation [16, 17]. Given the inhibitory effect of acacetin on the mitochondrial respiratory, the effect of acacetin on mROS production was investigated. MitoSOX staining showed that acacetin significantly inhibited mROS level in CD4+ T cells (Fig. 5B). The MMP maintains the activated state of CD4+ T cells [18], and the effect of acacetin on MMP was therefore determined in activated CD4+ T cells loaded with JC-1 fluorescence. Acacetin led to a significant reduction in MMP, as indicated by a decreased PE/FITC ratio (Fig. 5C). However, the impaired mitochondrial respiration was not due to a reduction in mitochondrial mass, since mitoTracker staining revealed that acacetin only mildly reduced mitochondrial mass at 10 μM (Fig. 5D). These results suggest that acacetin inhibits mROS generation and MMP in CD4+ T cells.

### Acacetin binds to mitochondrial complex II and inhibits its activity

**Fig. 6:**
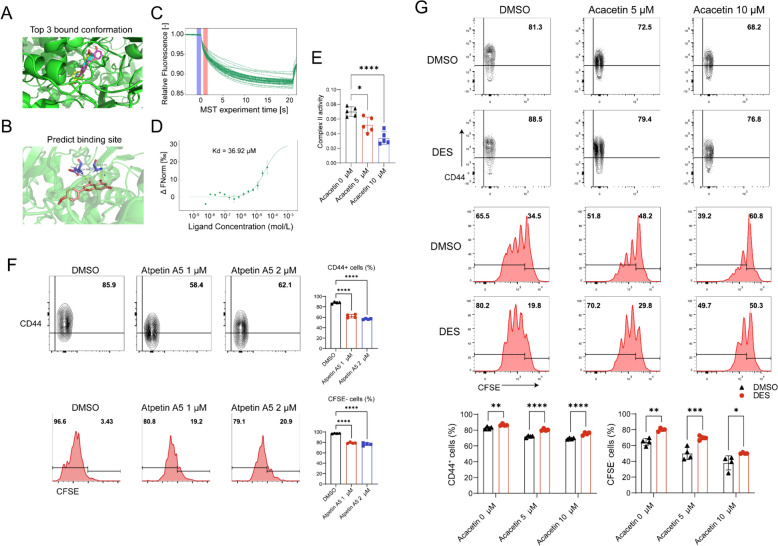
Molecular docking analyzes the interaction of acacetin with mitochondrial complex II. **A** The three lowest binding conformations of acacetin with complex II calculated by autodock-vina, represented by yellow, purple, and cyan, respectively, **B** Acacetin (red) bound to the amino acid residue sites of mitochondrial complex II (blue). **C** Normalized MST time-trace diagram of acacetin and SDHA protien. **D** MST S curve diagram, the Kd of combination is 36.92 μM.** E** The activity of complex II was detected in naive CD4+ T cells activated with anti-CD3/CD28 and cultured in the absence and presence of 5 or 10 μM acacetin for 48 h (n = 5). **F** Flow cytometry graphs and percent values of CD44 cells and CFSE cells determined in naive CD4+ T cells activated with anti-CD3/CD28 and cultured in the absence and presence of 1 or 2 μM Atpenin A5 for 72 h (n = 4). **G** Flow cytometry graphs and percent values of CD44 cells and CFSE cells determined in naive CD4+ T cells activated with anti-CD3/CD28 and cultured in the absence and presence of 5 or 10 μM acacetin and with or without DES for 72 h (n = 4). Data are expressed as mean ± SD and analyzed with one-way ANOVA followed by Tukey’s post hoc test (C and D) or two-way ANOVA followed by Bonferroni's post hoc test (E). *P < 0.05, **P < 0.01, ***P < 0.001, or ****P < 0.0001

The slight alteration in the mitochondrial mass of CD4+ T cells suggests that acacetin may modify the activity of the electron transport chain. The target of acacetin on the electron transport chain was predicted and analyzed using PharmMapper, a well-known pharmacophore matching and potential identification target platform [19]. Autodock-vina is a computational program that efficiently predicts the non-covalent binding of macromolecular receptors and small molecules and predicts the bound conformation and binding affinity with significantly improved prediction accuracy. According to the PharmMapper analysis, acacetin was found to bind to succinate dehydrogenase (z-score = 2.562), a component of mitochondrial complex II. The docking of acacetin to the complexes I-V using Autodock-vina showed that complex II had the lowest binding energy (Kcal/mol = − 9.2) compared to the other complexes (Table 2), and the first three binding conformations of complex II with acacetin had strong stability of RMSE, which indicates that the docking results are reliable (Fig. 6A, Table 3). Further analysis of the docked conformations with the lowest binding energies showed that akathisia can successfully bind to the subunit SDHA of complex II through hydrogen bonding interactions with GLU-398 and GLY-215 amino acid residues (Fig. 6B). To validate the interaction between acacetin and SDHA and to quantify the binding affinity, a MST assay was used. MST measures the movement of molecules in a microscopic temperature gradient field and detects changes in the hydration layer, charge, and size of molecules to quantify interactions between biomolecules. MST experiments confirmed the binding of acacetin to the complex II subunit SDHA protein, yielding a dissociation constant (Kd) of 36.92 μM (Fig. 6, C and D). Additionally, mutating the binding site of acacetin to SDHA resulted in a significantly decreased dissociation constant (Kd = 228.74 μM) (Figure S4). These results confirm the binding interaction between acacetin and SDHA.

**Table 2:** Binding energy of acacetin to complexes I-V predicting by autodock-vina

Number	Binding energy
Complex I	− 8.6
Complex II	− 9.2
Complex III	− 8.5
Complex IV	− 8.6
Complex V	− 7.5

**Table 3:** Top 10 conformations with minimal binding energy of acacetin to complex II

Number	Binding energy	RMSD
1	− 9.2	0
2	− 9.1	2.392
3	− 9	1.369
4	− 7.9	46.628
5	− 7.7	55.389
6	− 7.7	71.515
7	− 7.5	24.543
8	− 7.5	32.246
9	− 7.4	24.453

Mitochondrial complex II, also known as succinate dehydrogenase, is a central purveyor of the reprogramming of metabolic and respiratory adaptation in response to various intrinsic and extrinsic stimuli and abnormalities [20] and the complex II controls mROS and ATP production and regulates T cell activation [21, 22]. To corroborate the molecular docking findings, the impact of acacetin on complex II activity was assessed using a complex II activity assay kit. Acacetin was found to inhibit T-cell complex II activity in a dose-dependent manner (Fig. 6E). The effect of complex II activity on activation and proliferation was then determined by treating CD4+ T cells with the complex II inhibitor Atpenin A5 [23], and the results showed that activation and proliferation of CD4+ T cells were decreased by Atpenin A5 (Fig. 6F). Finally, DES, a membrane-permeable raw material of complex II was employed to test whether the function of CD4+ T cells could be restored by increasing the substrate of complex II. As expected, the decrease of CD44 expression and proliferation of CD4+ T cells by acacetin was reversed by application DES (Fig. 6G). These results suggest that acacetin inhibits CD4+ T cell mitochondrial function by affecting the complex II activity.

### Therapeutic administration of acacetin prevents the progression of DCM

**Fig. 7:**
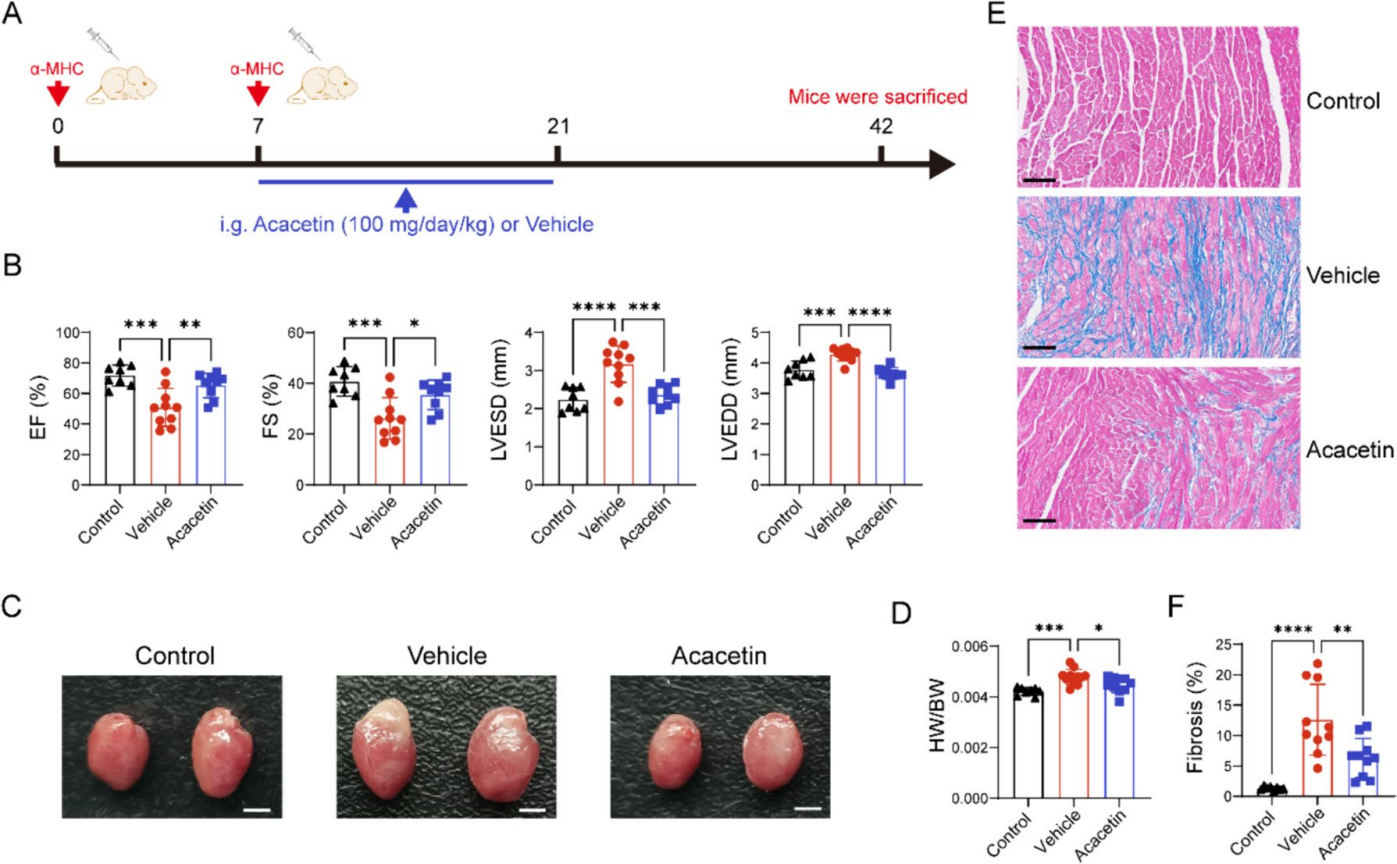
Therapeutic administration of acacetin decreases the progression of DCM in EAM mice. **A** Animal experimental protocol: α-MHC (0.2 mL) was subcutaneously injected at day 0 and day 7, and acacetin (100 mg/kg daily) or vehicle was orally administrated from day 7 to day 21 of the experiment. **B** Mean values of echocardiographic analysis for left ventricular EF, FS, LVESD, and LVEDD in control mice, EAM mice with vehicle and EAM mice with acacetin treatment (n = 8–10). **C** Representative heart images in mice as in B. Scale bar = 5 mm. **D** HW/BW of mice as in B (n = 8–11). **E** Representative images of masson stained ventricular tissue section slices (scale bar = 50 μm) in mice as in B. **F** Percent values of left ventricular fibrosis in mice as in B (n = 8–10). Data are expressed as mean ± SD and analyzed with one-way ANOVA followed by Tukey’s post hoc test. *P < 0.05, **P < 0.01, ***P < 0.001, or ****P < 0.0001

The beneficial effects of acacetin on EAM were observed through oral administration of the drug at the onset of the experimental procedure (Fig. 1A). However, most patients recovered from acute myocarditis may develop to DCM with myocardial fibrosis, ventricular dilatation, and heart dysfunction. Currently, there is no available drug in clinical practice to delay the onset of DCM. To investigate whether acacetin can delay and/or prevent the progression of DCM, acacetin was administered orally (100 mg/kg daily) for 2 weeks to EAM mice at day 7 post injection of myosin heavy chain-α peptides. The animals were sacrificed at day 42 of the experiment (Fig. 7A) after the heart function evaluation with echocardiography. Their hearts were isolated and weighted, and the myocardial sections were stained with Masson. The echocardiography analysis revealed that the left ventricular EF and FS were further reduced in EAM mice, compared to the results with the experiment of 21 days (Fig. 1C). Moreover, the left ventricular end-systolic diameter (LVESD) and left ventricular end-diastolic diameter (LVEDD), were greatly increased in EAM mice with vehicle, indicating that the DCM is developed to the heart failure during the experimental period of 42 days. It is interesting to note that the heart dysfunction and the increased LVESD and LVEDD were reversed in EAM mice with acacetin treatment (Fig. 7B, Table 4).

**Table 4:** Echocardiographic data in mice at day 42 of experiment

	Control	Vehicle	Acacetin
Heart rate (bpm)	437.8 ± 79.77	385.3 ± 26.28	415.6 ± 99.19
EF (%)	71.77 ± 6.88	50.80 ± 12.51^*^	65.16 ± 8.13^#^
FS (%)	40.73 ± 5.80	26.17 ± 8.16^*^	35.43 ± 5.86^#^
LV end-diastolic diameter	3.78 ± 0.29	4.28 ± 0.21^*^	3.66 ± 0.20^#^
LV end-systolic diameter	2.24 ± 0.31	3.17 ± 0.48^*^	2.36 ± 0.25^#^

Data show mean ± SD. P values were determined by one-way ANOVA followed by Tukey’s post hoc test

*EF* ejection fraction, *FS* fractional shortening, *LV* left ventricle

*P < 0.05 compared to Control, #P < 0.05 compared to Vehicle

In addition, the heart size and the ratio of HW/BW were increased in EAM mice with vehicle, but not in EAM mice with acacetin (Fig. 7C and D). The Masson-stained ventricular slices showed that great fibrosis observed in EAM mice with vehicle was significantly reduced in EAM mice with acacetin (Fig. 7E and F). These results indicate that administration of acacetin in acute phase of myocarditis can prevent the progression of DCM.

## Discussion

In this study, we demonstrate that acacetin inhibits cardiac injury and immune response in myocarditis and improves cardiac remodeling and ventricular dilation in DCM. The beneficial effects of acacetin on myocarditis are mainly related to inhibiting CD4+ T cell activation, proliferation and Th17 cell differentiation. Mechanically, acacetin inhibits CD4+ T cell activation by binding to and inhibiting the activity of mitochondrial complex II, thereby suppressing mitochondrial respiration, mROS production, and MMP. Our results suggest that acacetin is likely a novel drug candidate for treating myocarditis.

Acacetin has been shown as a promising drug candidate for treating various cardiovascular disorders, e.g. atrial fibrillation, myocardial infarction, atherosclerosis, etc., by targeting cardiomyocytes, endothelial cells, or macrophages [6, 7, 24]. Myocarditis is an inflammatory disease that is primarily driven by CD4+ T cell-mediated autoimmunity [25]. Myeloid cells and cardiomyocytes also contribute to the pathological process of myocarditis. We have previously demonstrated that acacetin inhibits the activation and proliferation of human T cells [11]. Another study has reported that acacetin reduces Th17 cells in mice with autoimmune arthritis [26]. The present study revealed that acacetin inhibited the development of myocarditis by suppressing CD4+ T cell activation and Th17 cell differentiation in vivo and in ex-vivo, which is consistent with previous reports. CD4+ T cells are key pathogenic factors in the development and progression of myocarditis. Several cytokines such as IFN-γ, IL-17A, IL-17F promote early tissue damage and progression of DCM of myocarditis. Acacetin can regulate dysregulated CD4+ T cell responses in myocarditis. Myeloid cells, including neutrophils, monocytes, and macrophages, have been shown to be involved in the myocarditis process [27]. An earlier study has reported that acacetin inhibits the activation and secretion of macrophages [28]. In this study, acacetin treatment mildly decreased the proportion of macrophages and monocytes in EAM mice, while the proportion of neutrophils was not altered. This suggests that acacetin modulates the myeloid cell response to some extent, but the detailed mechanism remains further studied.

Given our result showed that acacetin impacts T-cell activation starting from 5 μM rather than 2.5 μM, and exhibits a significant effect on T-cell activation, proliferation, and differentiation at 10 μM, we selected these two concentrations for RNA-seq to investigate the specific mechanism of acacetin's action on T cells. RNA-seq revealed that acacetin inhibited the expression of mitochondrial respiratory chain-related pathways and regulated cell cycling and lipid metabolism-related pathways. Mitochondria are the power plants that produce ATP. Mitochondrial respiration increases upon T-cell activation. Electron transfer from complex I to IV drives the proton gradient across the membrane, culminating in the production of ATP at complex V, which completes OXPHOS. OXPHOS is essential for T-cell activation, because the different complexes that inhibit the electron transport chain are sufficient to impair initial T-cell activation [29]. The results from this study confirmed that acacetin decreases mitochondrial respiration rate and ATP production of CD4+ T cells by inhibiting activation. mROS, one of important products of mitochondrial respiration, is also an indispensable part of T-cell signaling. mROS activates NFAT and subsequently induces IL-2 production, resulting in T-cell expansion [16]. MMP plays an important role in stabilizing mitochondrial morphology and function. In the present observation, acacetin inhibited mitochondrial respiration, mROS, and MMP in CD4+ T cells, indicating that acacetin inhibits T cell activation and proliferation through its action on mitochondrial function.

mROS are mainly produced by complexes I and III, and recently complex II has started to be considered as an alternative source [30, 31]. Succinate dehydrogenase forms mitochondrial complex II, which plays an important role in the electron transport chain and mitochondrial respiration. Studies have shown that inhibition of complex II activity inhibits mitochondrial respiration and thus affects T-cell proliferation and activation. Our molecular docking and MST assays confirm that acacetin binds to mitochondrial complex II and inhibits its activity. This was reinforced by reversion experiments with the addition of DES.

DCM is a major adverse outcome in patients with myocarditis. It is mainly associated with persistent inflammatory damage and autoantibody production. Our results show that the use of acacetin in the acute phase inhibits the progression of DCM. Persistent autoimmune CD4+ T cell-mediated immune responses and IL-17A secretion have been reported to be drivers of DCM progression. This study reveals that the beneficial effects of acacetin are primarily associated with reduction of CD4+ T cell- activity followed by reducing cardiomyocyte injury. Therefore, acacetin may have promising clinical application for treating myocarditis.

One limitation of this study is that acacetin acts in vivo not only on CD4+ T cells, but also on multiple targets such as macrophages, fibroblasts, and cardiomyocytes, in which detailed mechanisms remain to be clarified in the future studies.

## Conclusion

Collectively, the present study provides for the first time the new pharmacological effects that acacetin decreases the progression of myocarditis and cardiomyopathy via reducing mitochondrial complex II activity, thereby inhibiting mitochondrial respiration and mROS of CD4+ T cells, suggesting that acacetin may be a valuable therapeutic drug in CD4+ T cell-mediated myocarditis.

### Supplementary Information

Supplementary Material 1.

## Data Availability

The dataset supporting the conclusions of this article is available in the NCBI GEO database under accession number GSE221244.

## Abbreviations

DCM: Dilated cardiomyopathy
EAM: Experimental autoimmune myocarditis
RNA-seq: RNA sequencing
MST: Microscale thermophoresis
mROS: Mitochondrial reactive oxygen species
GEO: Gene Expression Omnibus
3D: Three-dimensional
EF: Ejection fraction
FS: Fractional shortening
HW/BW: Heart weight and body weight
PCA: Principal Component Analysis
LVESD: Left ventricular end-systolic diameter
LVEDD: Left ventricular end-diastolic diameter
