# Supplementary material for: Acacetin alleviates autoimmune myocarditis by regulating CD4+ T cell mitochondrial respiration
Source: Chin Med. 2024 May 13;19:68. doi: 10.1186/s13020-024-00943-9 (PMC11089761; doi:10.1186/s13020-024-00943-9)
Supplement: Supplementary file 1 — Supplementary Material 1. [file 13020_2024_943_MOESM1_ESM.docx]

**Supplementary material**

**Supplementary figure**


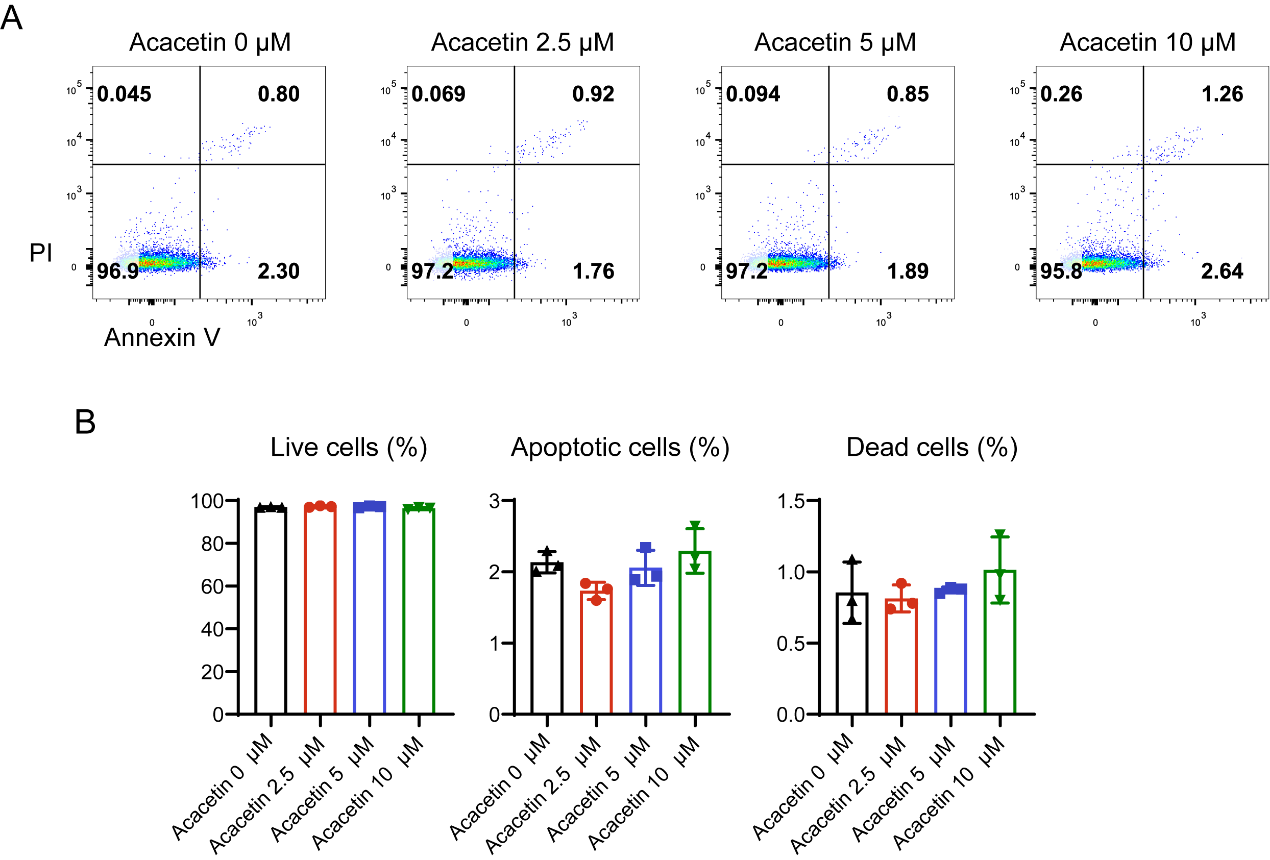


**Figure S1: Effect of acacetin on CD4+ T cell viability**

Naive CD4+ T cells from the spleen of normal mice were activated with anti-CD3/CD28 and cultured with different concentrations of acacetin for 72 h. Cells were stained with PI and Annexin V (AV). Representative flow cytometry graphs (A) and percent values (B) of live (PI-AV-), apoptotic (PI-AV+) or dead cells (PI+AV+), n=3/group. Results are representative of at least 2 independent experiments. Error bars show mean ± SD.


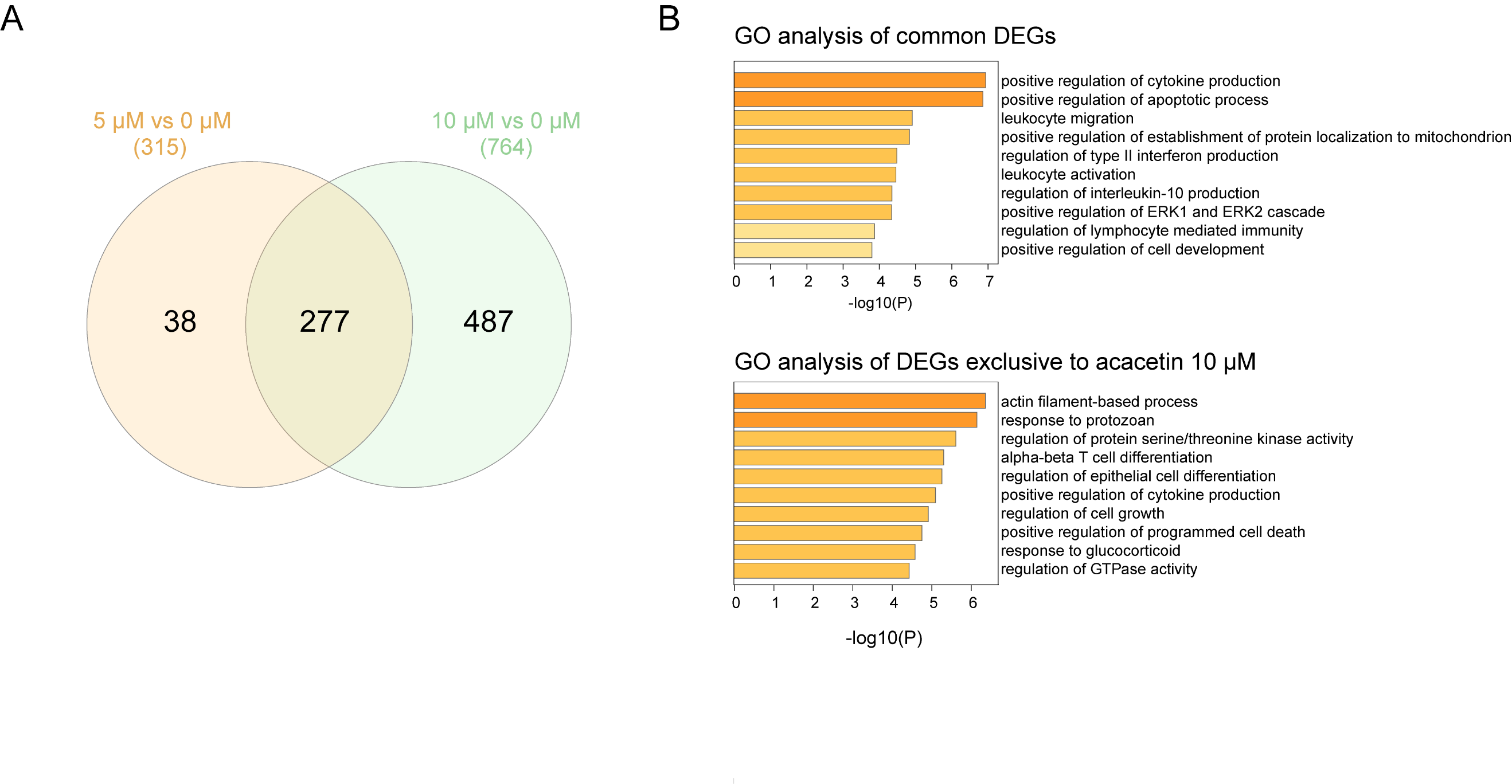


**Figure S2. Analysis of common DEGs**

A: Venn plot of DEGs of 5 μM and 10 μM of acacetin compared with 0 μM. B: Bar plot of GO analysis of common and exclusive to the 10 μM group of DEGs.


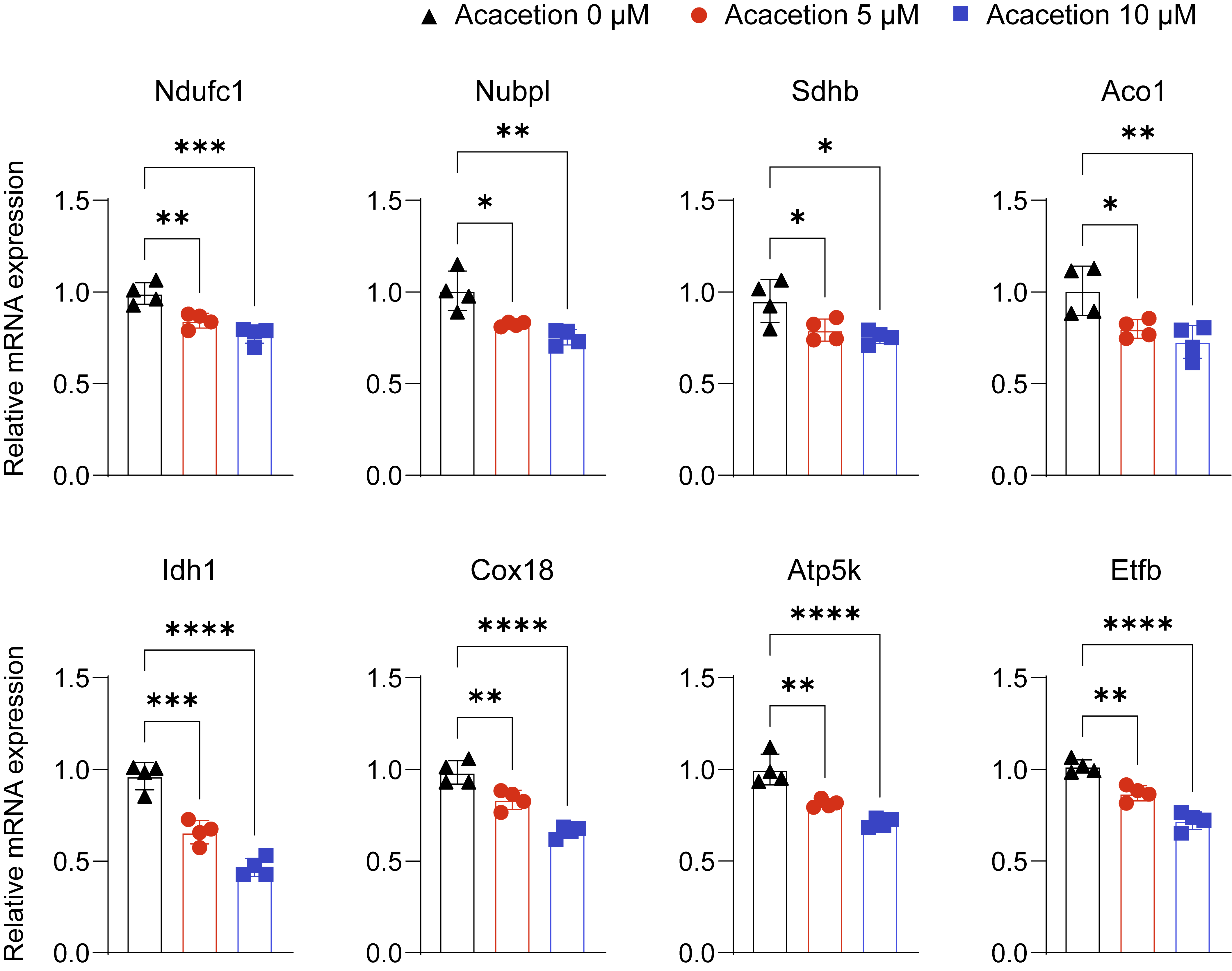


**Figure S3.** RT-qPCR detection of mitochondria-related gene expression in CD4+ T cells following 0, 5, and 10 μM acacetin treatment.


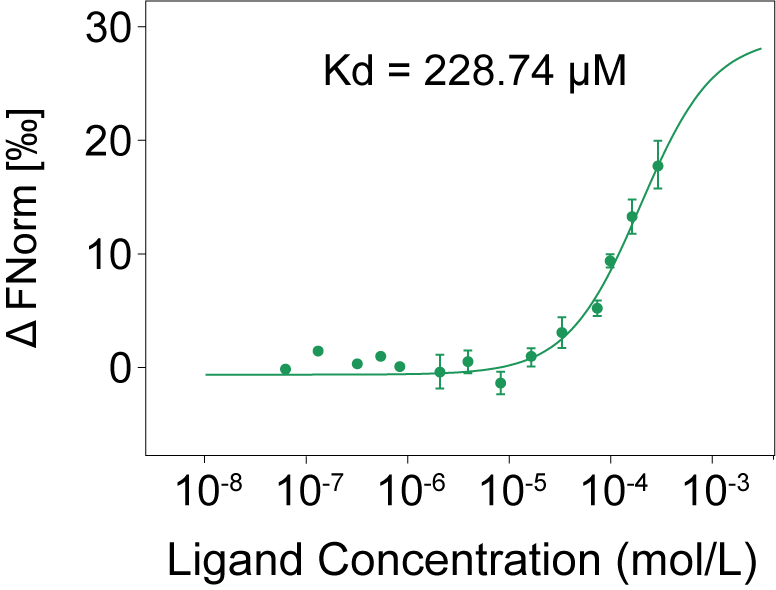


**Figure S4**. MST S curve diagram of the site-mutated SDHA protein and acacetin, the Kd of combination is 228.74 μM.

**Supplementary table**

Table S1 Primer sequences used for the RT-qPCR

| Gene name | Sequence 5’→3’ |
| --- | --- |
| *Il6* | Forward：ACAACCACGGCCTTCCCTACT  Reverse：CTCATTTCCACGATTTCCCAGA |
| *Il1b* | Forward：AAATACCTGTGGCCTTGGGC  Reverse：CTTGGGATCCACACTCTCCAG |
| *Tnf* | Forward：GCCTCTTCTCATTCCTGCTT  Reverse：TGGGAACTTCTCATCCCTTTG |
| *Ccl3* | Forward：TTCTCTGTACCATGACACTCTGC  Reverse：CGTGGAATCTTCCGGCTGTAG |
| *Ccl5* | Forward：GCTGCTTTGCCTACCTCTCC  Reverse：TCGAGTGACAAACACGACTGC |
| *Il17a* | Forward：TTTAACTCCCTTGGCGCAAAA  Reverse：CTTTCCCTCCGCATTGACAC |
| *Il17f* | Forward：TGCTACTGTTGATGTTGGGAC  Reverse：AATGCCCTGGTTTTGGTTGAA |
| *Ifng* | Forward：ATGAACGCTACACACTGCATC  Reverse：CCATCCTTTTGCCAGTTCCTC |
| *Ndufc1* | Forward：GTAGTGCTGCGCTCGTTTTC  Reverse：CCAACCAGTTAGGTTTGGCAT |
| *Nubpl* | Forward：GTTGGCTTGTTAGATGTGGATGT  Reverse：GCGCAGTCTCTTCAACCAAAA |
| *Sdhb* | Forward：AATTTGCCATTTACCGATGGGA  Reverse：AGCATCCAACACCATAGGTCC |
| *Aco1* | Forward：AGAACCCATTTGCACACCTTG  Reverse：AGCGTCCGTATCTTGAGTCCT |
| *Idh1* | Forward：ATGCAAGGAGATGAAATGACACG  Reverse：GCATCACGATTCTCTATGCCTAA |
| *Cox18* | Forward：GGCCGCCTATCAGCACTAC  Reverse：TGAGATAAGTCTCCGCATGTTCT |
| *Atp5k* | Forward：GTTCAGGTCTCTCCACTCATCA  Reverse：CGGGGTTTTAGGTAACTGTAGC |
| *Etfb* | Forward：CTGTCAAGAGGGTCATCGACT  Reverse：CACAGAAGGGGTTCATGGAGT |
| *Gapdh* | Forward：CTGGGCTACACTGAGCACC  Reverse：AAGTGGTCGTTGAGGGCAATG |

Table S2 analysis of common DEGs

| [5μm vs 0μm] and [10μm vs 0μm] | [5μm vs 0μm] | [10μm vs 0μm] | [10μm vs 0μm] |
| --- | --- | --- | --- |
| Trp53inp1 | Treml2 | Lad1 | Rgmb |
| Nr4a2 | Slc35d3 | Ccr7 | Rnf208 |
| Cish | P2ry1 | Satb1 | Ier3 |
| Lgals1 | Ciart | Cdkn1a | Slc7a3 |
| Cd69 | Fndc9 | Gimap3 | Cpt1b |
| Sik1 | Me3 | Ptp4a3 | Il10 |
| Jun | Zfp329 | Ddit4 | Sgk1 |
| Sell | Adssl1 | Nfkbia | Kcnk10 |
| Zbtb32 | Kcnc3 | Grap | Nuak2 |
| Irf2bp2 | Tnfrsf23 | Ikbke | Lypd6b |
| Tagap | Trav14n-1 | Pim1 | C130036L24Rik |
| Myh10 | Phtf1os | Tnfaip3 | Smug1 |
| Phlda3 | H3c3 | Zfp862-ps | Bloc1s3 |
| Bbc3 | Nbdy | Tnfsf8 | Pagr1a |
| Ikzf3 | Zmat1 | Niban1 | Iqsec2 |
| Angptl2 | BC051077 | Ms4a4b | Mcoln3 |
| Trib2 | Csf2rb2 | Psrc1 | Tex14 |
| Dsp | Map2 | H1f0 | Spock2 |
| Ccl3 | Pcdhga10 | Ttyh3 | Myo1h |
| Plk2 | mt-Nd3 | Stat5a | Dynlt2a2 |
| Cadm1 | Grk4 | Dusp1 | Lurap1 |
| Eno2 | Zfp968 | Il2 | Lgr4 |
| Tmem63b | Fam160a1 | Zfp36l1 | Ptgr1 |
| Chst2 | Nectin2 | Gvin2 | Mylip |
| Hmcn2 | BC043934 | Hmgcs1 | St3gal5 |
| Fam169b | Rpsa-ps2 | Irf8 | Tbc1d8 |
| Slco4a1 | Gpr137b | Pdcd1 | Gas2 |
| Plek | Dkkl1 | Btg1 | Cdc42bpa |
| Amigo2 | Stk32c | Zfp36 | Sema6b |
| Enc1 | Aif1l | Psd | Ifit1 |
| Abcd3 | Phospho1 | Sesn2 | Siglece |
| Col5a3 | Ptprk | Bcl2l1 | Abcb10 |
| Ifitm3 | Mfrp | Mapre2 | C1ra |
| Ypel3 | Tmem265 | Nfkbiz | Tbx2 |
| Klhl24 | Rpl17-ps8 | Rnf19a | Nkd2 |
| Ptpn5 | Gmpr | Ltb | Egr4 |
| Ccl4 | Fam124a | Zan | Zfp976 |
| Pik3ip1 | Pik3r3 | Tgtp1 | Cpne7 |
| Lgals7 |  | Tgtp2 | Zfp85 |
| Ptger4 |  | Slc19a2 | Nppc |
| Pros1 |  | Pidd1 | Rapgef5 |
| Ccr4 |  | Spry1 | Nqo1 |
| Sectm1a |  | Ier5 | Zfp433 |
| Cdc42bpg |  | Pnrc1 | mt-Nd4l |
| Tnnt2 |  | Thy1 | Rgs9 |
| Dgkk |  | B4galt1 | Myo1e |
| Mlkl |  | Lif | Usp35 |
| Tnfrsf13c |  | Gnptab | Sdc1 |
| Tnfsf4 |  | Gata3 | Hsh2d |
| Nkg7 |  | Cep170b | Pdzrn3 |
| Nos2 |  | Slc16a1 | Tnip3 |
| Ubash3b |  | Sft2d2 | E130208F15Rik |
| Inka2 |  | Tecpr1 | Arhgef40 |
| Polk |  | Ifih1 | Arhgef25 |
| Tnfrsf26 |  | Glipr1 | Parp12 |
| Crebrf |  | Plk1 | C1qtnf5 |
| Vipr1 |  | Mapkapk3 | Macroh2a2 |
| Ramp3 |  | Slfn1 | Tlcd2 |
| Ly6a |  | Dennd5a | Dusp18 |
| Atf5 |  | Fbxl20 | Ptpn14 |
| Dclk1 |  | Als2cl | Oas2 |
| Ddit3 |  | Gbp7 | Zfp629 |
| Cth |  | Tulp4 | Pdk2 |
| Tigit |  | Eif2ak3 | Micall2 |
| Serpina3g |  | Orai2 | Fbxo48 |
| Ikzf4 |  | Mmd | Pwwp4a |
| Filip1l |  | Iigp1 | Igsf9 |
| Hpgds |  | Npas4 | Tet1 |
| Akr1c18 |  | Bcl2 | H2ac19 |
| Sema4c |  | Zhx2 | Gab2 |
| Fscn1 |  | Fam102a | Prob1 |
| Pmaip1 |  | Arap2 | Ltbp3 |
| Tbx21 |  | Flcn | Prr7 |
| Tbkbp1 |  | Map3k8 | Grin2d |
| Acsl6 |  | Pck2 | Clic3 |
| Mt1 |  | Plxna1 | Ppp1r3f |
| Robo3 |  | Rnf169 | Zfp661 |
| Mafb |  | Lap3 | Twist1 |
| Ehd2 |  | Snhg15 | Ahnak2 |
| Crabp2 |  | Mcoln1 | Tns4 |
| Pla2g4f |  | Lpin1 | Arhgap23 |
| Etv4 |  | Lrrc28 | Pdgfrb |
| Serpina3f |  | Vat1 | Zbtb46 |
| Casz1 |  | Gramd3 | Perm1 |
| E430014B02Rik |  | Mxd4 | Rtl5 |
| Nr1d2 |  | Bsdc1 | Tceal8 |
| Sulf2 |  | Mxd1 | Ighg1 |
| Cyp1a1 |  | Dusp2 | Serpinc1 |
| Zbtb10 |  | Btg2 | Ttbk1 |
| Lancl3 |  | Klf3 | Ctsf |
| Olfr1372-ps1 |  | Atg9b | Dnah14 |
| Cacnb3 |  | Hemk1 | Opn3 |
| Eci1 |  | Tjp2 | Kifc2 |
| Gprin3 |  | Serinc5 | Mtfp1 |
| Cpm |  | Calcoco1 | Dixdc1 |
| Trib3 |  | Ulk1 | Padi2 |
| Try5 |  | Ifngr2 | Gpr179 |
| Insl6 |  | Fndc10 | Slc66a1 |
| Socs2 |  | Snx20 | Igflr1 |
| Farp1 |  | Tmem38b | Arhgap29 |
| Slc6a9 |  | Crtam | Slc16a5 |
| Zfp750 |  | Pisd-ps1 | Fap |
| Galm |  | Arl4c | R74862 |
| Adam19 |  | Brpf3 | Cd72 |
| Palm3 |  | Klf4 | Podn |
| Il3 |  | Rgs2 | Marveld2 |
| Ppfibp2 |  | Hbp1 | Npl |
| Fads2 |  | Lrif1 | Cyp11a1 |
| Crebl2 |  | Tnfrsf10b | Ccr5 |
| Ccdc141 |  | Rgs12 | Tmem140 |
| Akr1b8 |  | Rnf167 | Ppp1r32 |
| E430024P14Rik |  | Igsf23 | D130007C19Rik |
| Itga3 |  | Ptger2 | Wdr66 |
| Mlf1 |  | Dnah8 | Rpl17-ps10 |
| Trat1 |  | Vrk3 | Dock3 |
| Klrk1 |  | Ip6k2 | Fgd1 |
| Itga9 |  | Arhgef9 | Spef2 |
| S1pr1 |  | Ift80 | Gnat1 |
| Tnfsf9 |  | Mcoln2 | Emilin2 |
| Gcat |  | Tbc1d17 | Lilr4b |
| Mt2 |  | Gzmb | Pls1 |
| Rpph1 |  | Tnf | Adgrd1 |
| D130062J10Rik |  | Ccr8 | Sftpb |
| Srxn1 |  | Stat4 | Ppp1r26 |
| Srgap3 |  | Ddr1 | Fam171a1 |
| Niban2 |  | Vegfa | Asah2 |
| Cnr2 |  | Ypel2 | Slc37a2 |
| Fhl2 |  | Bet1l | Pard3 |
| Zkscan7 |  | Bmp1 | Zfp970 |
| Chac1 |  | Spata7 | Actn2 |
| Prr33 |  | Endod1 | Dmxl2 |
| Lair1 |  | Mpzl3 | Map2k3os |
| Rragd |  | BE692007 | Plppr2 |
| Dnah7a |  | Slc25a24 | Kctd15 |
| Capza1-ps1 |  | Id3 | Map3k9 |
| Cxcr3 |  | Tnfaip8 | Ptgs2 |
| Lilrb4a |  | N4bp2l1 | Jcad |
| Maoa |  | Gadd45g | Cdhr4 |
| Ahrr |  | Cd55 | Fbn2 |
| Cxcr4 |  | Oas3 | Bbs10 |
| Shisa8 |  | Mirt1 | Slc30a2 |
| Arl4d |  | Maff | Peli3 |
| Mpzl2 |  | Arrdc4 | Depdc7 |
| Zfp874a |  | Lratd2 | Pcyt1b |
| Zfp385a |  | Plin2 | Grb10 |
| Fbxo32 |  | Smox | Zbtb26 |
| Coq8a |  | Irgq | Lima1 |
| Eomes |  | Smad7 | Omd |
| Snx8 |  | Sntb1 | Ak9 |
| B630019A10Rik |  | Zcwpw1 | Celf5 |
| Nt5e |  | Dnm3 | Sbsn |
| Ddit4l |  | Plxna3 | Tmem35a |
| Pak1 |  | Adap1 | Olfr1033 |
| Igf2bp3 |  | Bcl3 | Zfp174 |
| Dact3 |  | Cyp4f13 | Ggt5 |
| Dusp8 |  | Elovl6 | Prkar2b |
| Pdgfa |  | Dnph1 | Nipal1 |
| Il12rb2 |  | Atp1b1 | Setbp1 |
| Amt |  | Pkn3 | E130102H24Rik |
| D630039A03Rik |  | Prickle3 | Numbl |
| Gria3 |  | Zfp688 | Plpp1 |
| Ckap4 |  | Izumo4 | Trim34b |
| Fam171b |  | Cpne2 | Slc6a13 |
| Apbb1 |  | Pink1 | Nat14 |
| Shtn1 |  | Zc3h6 | Usp44 |
| C3 |  | Ccdc28a | Kash5 |
| Xcl1 |  | Whrn | Cyp2f2 |
| Zbtb20 |  | Rora | Sema6c |
| Bok |  | Vmac | C230037L18Rik |
| Cd160 |  | Jazf1 | Mterf1b |
| Lhfp |  | H2-T24 | Tspan6 |
| Slamf9 |  | Cd83 | Col5a2 |
| Cdk5r1 |  | Mgmt | Klhl23 |
| Marcks |  | Tcp11l2 | Bdh2 |
| Ikzf2 |  | C030037D09Rik | Nova2 |
| Cavin3 |  | Slc25a13 | Vat1l |
| Ankrd39 |  | Klhl42 | Zfr2 |
| Kantr |  | Ccrl2 | Cav2 |
| Wnt3 |  | Pard6g | C230085N15Rik |
| Cox6b2 |  | C9orf72 | Catsperd |
| Olfml2a |  | Chaserr | Rpp25 |
| Ccdc184 |  | Zrsr1 | Chac2 |
| Gpr55 |  | Gbp10 | Stab1 |
| Lipg |  | Usp46 | Vwa1 |
| Nrg3 |  | Atp2b4 | Zfp459 |
| Wscd2 |  | Ctns | Sphk1 |
| Spint1 |  | Zbtb37 | Klhdc7a |
| Hip1 |  | Dyrk1b | Ankrd33b |
| Cd70 |  | Il7r | Ccdc102a |
| Rassf8 |  | Gk | Carmil1 |
| Nyx |  | Rai14 | Prss16 |
| B930095G15Rik |  | Pcnx4 | Dlx1 |
| Cpt1c |  | Slc1a4 | Map2k6 |
| Frmpd1 |  | Colq | Atp8a2 |
| Tspan2 |  | Dcxr | Myo1a |
| P3h2 |  | Mapk1ip1 | S100a3 |
| Ccdc159 |  | Cdh23 | Scn3b |
| Oas1b |  | Sfn | Ccl6 |
| Stra6 |  | Abcb4 | B230206L02Rik |
| L1cam |  | Hid1 | Id1 |
| Fasl |  | Tns1 | Upb1 |
| Fgr |  | Nebl | Pgam2 |
| Mpeg1 |  | P2rx7 | Cass4 |
| Cracdl |  | Pik3ap1 | Sncg |
| Tnp2 |  | Adrb2 | Hba-a1 |
| Isg15 |  | Mns1 | Wnk2 |
| Scin |  | Slc25a45 | Nkain1 |
| Plxdc1 |  | Kbtbd12 | Ggn |
| Aipl1 |  | Utp14b | Cnrip1 |
| C1s2 |  | Niban3 | Ninl |
| Dnah12 |  | Ckmt1 |  |
| Tbx6 |  | Casp4 |  |
| Tmem121b |  | Epsti1 |  |
| Sh3bgrl2 |  | Repin1 |  |
| Bag2 |  | Wfikkn1 |  |
| Fam167a |  | Serpine1 |  |
| Lrrc32 |  | Ube2l6 |  |
| Nts |  | Chrm4 |  |
| Dhrs9 |  | Tnfsf10 |  |
| Mreg |  | Xdh |  |
| Casp1 |  | Pglyrp2 |  |
| Fam83h |  | Acrbp |  |
| Mcc |  | Slc49a4 |  |
| Rsph1 |  | Ptpdc1 |  |
| Ascl4 |  | Rab31 |  |
| Chit1 |  | Ankrd24 |  |
| Bspry |  | Igfbp7 |  |
| Fam222a |  | Etfbkmt |  |
| Il18rap |  | Etl4 |  |
| Slc13a3 |  | Abcg1 |  |
| Vax2 |  | Evi2a |  |
| Ifi208 |  | Perp |  |
| Adm |  | Rasgrp2 |  |
| Dnhd1 |  | Dock7 |  |
| Fbxo24 |  | Gpt2 |  |
| Csgalnact1 |  | Cfl2 |  |
| Mctp1 |  | Nupl1 |  |
| Sp6 |  | Tgfbr3l |  |
| Rnf128 |  | Zfp358 |  |
| Hspa1l |  | Spire1 |  |
| Rbpms2 |  | Tesk2 |  |
| Serpinf1 |  | Ttc28 |  |
| Nqo2 |  | Lysmd4 |  |
| Stap2 |  | Plcd3 |  |
| Fbxo2 |  | Myo6 |  |
| Klrb1f |  | Mycbp |  |
| Apol8 |  | Sap25 |  |
| Stmn4 |  | Ryr2 |  |
| Pcdh7 |  | C1s1 |  |
| Cd40 |  | Krt16 |  |
| Samd11 |  | Trim7 |  |
| Notch3 |  | Tex21 |  |
| Bcas1 |  | Cdh15 |  |
| Arhgdig |  | Ttll10 |  |
| Bcl2l14 |  | Itga2b |  |
| Apol10b |  | Specc1 |  |
| Dbp |  | Cdo1 |  |
| H3c13 |  | Letm2 |  |
| Car5b |  | Zfp784 |  |
| Frmd4a |  | Dcun1d3 |  |
| Cd209c |  | Sdc4 |  |
| Otx1 |  | Rhod |  |
| D5Ertd605e |  | Dock1 |  |
| Clcnkb |  | Frmd4b |  |
| Tm4sf5 |  | Bend5 |  |
| Prelid3a |  | Fsbp |  |
| Degs2 |  | Cxcr5 |  |
| Kazn |  | Dennd3 |  |
| Unc13b |  | Siglecf |  |
| BC106179 |  | Dlg2 |  |
| H3c15 |  | Rps2-ps8 |  |
| Rdh5 |  | E130307A14Rik |  |
| Abca1 |  | Kcnh2 |  |
| Slc17a6 |  | Gpr137b-ps |  |
| Tcp11l1 |  | Fgfr3-ps |  |
| Selp |  | Cercam |  |
| Dmrta2 |  | Dock5 |  |
